# Antiatherosclerotic effect of dehydrocorydaline on ApoE^−/−^ mice: inhibition of macrophage inflammation

**DOI:** 10.1038/s41401-021-00769-3

**Published:** 2021-09-22

**Authors:** Bin Wen, Yuan-ye Dang, Su-hua Wu, Yi-min Huang, Kong-yang Ma, Yi-ming Xu, Xi-Long Zheng, Xiao-yan Dai

**Affiliations:** 1 Guangdong Provincial Key Laboratory of Molecular Target & Clinical Pharmacology, School of Pharmaceutical Sciences, Guangzhou Medical University, Guangzhou, 511436 China; 2 China Centre for Infection and Immunity Studies (CIIS), School of Medicine, Sun Yat-sen University, Shenzhen, 518107 China; 3 School of Basic Medical Sciences, Guangzhou Medical University, Guangzhou, 511436 China; 4 Department of Biochemistry & Molecular Biology, Cumming School of Medicine, University of Calgary, Calgary, AB Canada

**Keywords:** atherosclerosis, dehydrocorydaline, macrophage, inflammation

## Abstract

Despite improvements in cardiovascular disease (CVD) outcomes by cholesterol-lowering statin therapy, the high rate of CVD is still a great concern worldwide. Dehydrocorydaline (DHC) is an alkaloidal compound isolated from the traditional Chinese herb *Corydalis yanhusuo*. Emerging evidence shows that DHC has anti-inflammatory and antithrombotic benefits, but whether DHC exerts any antiatherosclerotic effects remains unclear. Our study revealed that intraperitoneal (i.p.) injection of DHC in apolipoprotein E-deficient (ApoE^−/−^) mice not only inhibited atherosclerosis development but also improved aortic compliance and increased plaque stability. In addition, DHC attenuated systemic and vascular inflammation in ApoE^−/−^ mice. As macrophage inflammation plays an essential role in the pathogenesis of atherosclerosis, we next examined the direct effects of DHC on bone marrow-derived macrophages (BMDMs) in vitro. Our RNA-seq data revealed that DHC dramatically decreased the levels of proinflammatory gene clusters. We verified that DHC significantly downregulated proinflammatory interleukin (IL)-1β and IL-18 mRNA levels in a time- and concentration-dependent manner. Furthermore, DHC decreased lipopolysaccharide (LPS)-induced inflammation in BMDMs, as evidenced by the reduced protein levels of CD80, iNOS, NLRP3, IL-1β, and IL-18. Importantly, DHC attenuated LPS-induced activation of p65 and the extracellular signal-regulated kinase 1/2 (ERK1/2) pathway. Thus, we conclude that DHC ameliorates atherosclerosis in ApoE^−/−^ mice by inhibiting inflammation, likely by targeting macrophage p65- and ERK1/2-mediated pathways.

## Introduction

Atherosclerosis is a leading cause of mortality and morbidity worldwide because of its pathological roles in most cardiovascular diseases (CVDs), such as coronary artery disease, myocardial infarction, and stroke [1]. Under chronic inflammatory pathological conditions, atherosclerosis gradually develops from fatty streaks into atherosclerotic plaques in the arterial intima [2]. The independent risk factors for atherosclerosis include high blood cholesterol, high blood pressure, and diabetes mellitus, which directly activate circulatory monocytes and upregulate the expression of adhesion molecules and chemokines in endothelial cells, leading to the recruitment of monocytes (usually inflammatory Ly6C^hi^ monocytes) to the artery wall [3, 4]. During the pathogenesis of atherosclerosis, these monocytes differentiate into macrophages, which take up a large amount of lipids and become macrophage-derived foam cells [5]. Foam cells secrete large amounts of inflammatory cytokines such as tumor necrosis factor α (TNFα), interleukin 6 (IL-6), IL-1β, matrix metalloproteinases (MMPs) such as MMP-9, and chemokine (C-C motif) ligand 2 (CCL2), which exacerbate vascular inflammation and reduce plaque stability [6]. Increasing evidence has indicated a critical role for inflammatory macrophage activation in atherosclerosis initiation and development, suggesting that modulating macrophages may be a promising strategy for atherosclerosis intervention.

To date, a well-accepted treatment of atherosclerosis is the use of lipid-lowering drugs, such as statins [7]. It is important to note that statins induce severe side effects, including myopathy, gastrointestinal disorders, renal damage, liver function impairment, and fatigue, and do not improve other subclinical atherogenic risk factors [8]. Thus, alternative effective treatments have been under intensive development. In the Canakinumab Anti-inflammatory Thrombosis Outcomes Study (CANTOS) trial, for example, the use of the anti-IL-1β monoclonal antibody canakinumab as an anti-inflammatory treatment was shown to reduce the rate of recurrent cardiovascular events [9], suggesting that inflammation is a novel therapeutic target for atherosclerosis and associated diseases.

Dehydrocorydaline (DHC), an alkaloid isolated from *Corydalis yanhusuo*, was initially demonstrated to prevent norepinephrine release from adrenergic nerve terminals in both the taenia cecum and pulmonary arteries [10]. DHC was then shown to have potent anti-inflammatory effects on acute and chronic animal models [11]. Since DHC has anti-inflammatory functions, it is widely used in the treatment of inflammation-associated pain [12], such as cramping pain, abdominal pain, and pain caused by injury [13], and bone cancer [14]. With respect to the cardiovascular effects of DHC, it decreases intracellular free calcium concentrations in cardiomyocytes under normoxia and hypoxia, suggesting an inhibitory effect on calcium overload [15]. Importantly, DHC has an antispasmodic effect on coronary arteries and may play a therapeutic role in coronary heart disease [16]. In summary, DHC shows anti-inflammatory and cardioprotective effects. However, whether DHC has any direct effect on the development of atherosclerosis remains largely unknown.

In the present study, we have shown that DHC attenuates atherosclerosis and reduces systemic and vascular inflammation in apolipoprotein E-deficient (ApoE^−/−^) mice. Our RNA sequencing (RNA-seq) results reveal that DHC drastically inhibits various inflammatory responses in macrophages. Specifically, DHC decreases lipopolysaccharide (LPS)-induced expression of various proinflammatory mediators by repressing p65 and extracellular signal-regulated kinase 1/2 (ERK1/2) activation in macrophages. Our findings suggest that DHC is a novel therapeutic candidate for the treatment of atherosclerotic CVD.

## Materials and methods

### Chemicals and reagents

DHC was purchased from Chroma-Biotechnology (30045-16-0, Chengdu, China) and dissolved in dimethyl sulfoxide (DMSO; Sigma-Aldrich). DHC was prepared for intraperitoneal (i.p.) injection formulas as follows: 1% DHC + 30% PEG30 (Selleck, S6704-100g) + 5% Tween 80 (Selleck, S6702-100mL) + 64% H_2_O. Optimal cutting temperature compound (OCT) was obtained from Leica (Wetzlar, Germany).

### Animals and treatments

Male 8-week-old ApoE^−/−^ mice were purchased from Beijing HFK Bioscience Company (Beijing, China). A Western diet (0.15% cholesterol and 21% fat) was obtained from Guangdong Medical Laboratory Animal Center. ApoE^−/−^ mice were randomly assigned to the vehicle (i.p. injection of DMSO, daily) or DHC (i.p. injection of 5 mg/kg DHC, daily) groups. All mice were fed a Western diet for 12 weeks, given *ad libitum* access to water, and kept on a 12 h light–dark cycle. All mice were housed in the specific pathogen-free facility, and no mice was excluded from analysis. All animal experiments were approved by the Animal Research Ethics Committees of Guangzhou Medical University.

### Cell culture

As previously described, bone marrow-derived macrophages (BMDMs) were isolated from C57BL/6J mice [17]. Cells were cultured in RPMI-1640 (HyClone, SH30809.01) medium containing 10% fetal bovine serum (FBS, Gibco, 10091-148-500 ml), 1% penicillin/streptomycin (Gibco, 15140122), and recombinant murine M-CSF protein (25 ng/mL, eBioscience, 14-8983-80) and maintained at 37 °C in 5% CO_2_. Ultrapure LPS (InvivoGen, tlrl-smlps) and IFNγ (mouse) recombinant protein (Invitrogen, RP-8617) were used to induce an inflammatory response in BMDMs.

### Cell counting kit-8 (CCK-8) assay

The cytotoxicity of DHC was evaluated using BMDMs seeded into 96-well plates. Cells were treated with 0, 10, 25, 50, 100, and 200 μM DHC for 24 h and then incubated with CCK-8 (YEASEN, 40203ES76) working solution at 37 °C for 2 h. The optical density was measured at 450 nm using a microplate reader, and viable cells were calculated.

### RNA-seq analysis

Total RNA was extracted from DMSO- and DHC-treated BMDMs (*n* = 3). Transcriptome sequencing experiments were performed by Majorbio Bio-Pharm Technology (Shanghai, China). Briefly, an RNA-seq transcriptome library was prepared according to the TruSeq^TM^ RNA sample preparation kit from Illumina (San Diego, CA, USA) using 1 μg of total RNA. After quantification by TBS380, the paired-end RNA-seq sequencing library was sequenced with the Illumina NovaSeq 6000 System (2 × 150 bp read length). Genes with significantly different expression levels (*P* adjusted < 0.05 and | log2FC | ≥ 1) were selected for further processing. Kyoto Encyclopedia of Genes and Genomes (KEGG) analyses were performed to identify differentially expressed genes (DEGs) that were significantly enriched in the pathways at Bonferroni-corrected *P* ≤ 0.05 compared with the whole-transcriptome background. KEGG pathway analyses were carried out by KOBAS (http://kobas.cbi.pku.edu.cn/home.do) [18].

### Aortic ultrasonography

Doppler ultrasound (Vevo 2100, FUJIFILM VisualSonics, Canada) was used to perform ultrasound imaging of mice. The mice were anesthetized with isoflurane (RWD, #R51022) and then placed in the supine position on a temperature- and electrocardiogram-controlled plate. The heart rates of the mice were maintained at 400–450 beat/min. B-mode imaging was used to observe atherosclerotic plaques, and the peak diastolic velocity (PDV) of the aorta was calculated.

### Imaging

Aortas were obtained from ApoE^−/−^ mice fed a western diet for 12 weeks. The isolated aorta was immediately immersed in OCT and frozen for further experiments. The atherosclerotic lesion area at the aortic root was analyzed by preparing cross-sections (6 μm) and placing them on glass slides. These six serial cryosections from each mouse were fixed in 4% paraformaldehyde (Servicebio, G1101) and stained with oil red O solution (Sigma-Aldrich, O0625). Hematoxylin and eosin (H&E) staining (Beyotime, C0105) and Masson’s trichrome (Solarbio, G1346) staining were performed for morphometric lesion analysis according to the manufacturers’ instructions. Images of atherosclerotic plaques were captured by an Aperio Digital Pathology Slide Scanner (Aperio CS2), and quantification was performed using ImageJ software.

### Immunofluorescence staining

Aortic root sections were fixed in ice-cold acetone for 10 min, washed with phosphate-buffered saline (PBS), and blocked with normal goat serum for 1 h at room temperature, after which the sections were incubated with primary antibodies (anti-F4/80, 1:200, CST, 30325; anti-α-SMA, 1:100, Abcam, ab5694) at 4 °C overnight. The sections were rinsed with PBS, incubated with secondary antibodies for 1 h at room temperature, and then mounted with flourished mounting medium containing 4′,6-diamidino-2-phenylindole (DAPI). Images were captured by a Nikon A1R confocal microscope. The F4/80^+^ and α-SMA^+^ areas (normalized to the lesion) were assessed using maximal intensity projection images, and the analysis was performed using ImageJ software. The vulnerability index was calculated using the following formula: (macrophage staining% + lipid staining%)/(VSMC staining% + collagen staining%) [19].

### Enzyme-linked immunosorbent assay (ELISA)

Plasma cytokines were quantified using ELISA kits for mouse TNFα (MTA00B, R&D Systems) and IL-1β (MLB00C, R&D Systems) according to the manufacturer’s instructions.

### Plasma lipid examination

Low-density lipoprotein cholesterol (LDL-C) (Gcell, GS141Z), high-density lipoprotein cholesterol (HDL-C) (Gcell, GS131Z), total cholesterol (TC) (Gcell, GS101Z) and triglycerides (TG) (Maccura, CH0101151) were measured by a Hitachi 7600 automatic biochemical analysis instrument.

### Western blotting

BMDMs were lysed in RIPA buffer with a protease inhibitor cocktail (MedChemExpress, HY-K0010), and the protein concentrations were measured by the BCA protein assay (Beyotime, P0012). The protein lysates were resolved by SDS–PAGE and transferred onto nitrocellulose (NC) membranes. The membranes were blocked with 5% skimmed milk for 1 h at room temperature and then incubated with primary antibodies and HRP-conjugated secondary antibodies. The primary antibodies were as follows: CD80 (Abcam, ab238481), iNOS (Thermo Fisher, PA1-036), NLRP3 (Life Science, AG-20B-0014-C100), IL-1β (R&D Systems, AF-401-NA), IL-18 (Abcam, ab71495), p-p65 (Cell Signaling Technology, 3033), p65 (Cell Signaling Technology, 8242), p-ERK1/2 (Cell Signaling Technology, 4376), ERK1/2 (Cell Signaling Technology, 9102), p-p38 (Cell Signaling Technology, 9215), p38 (Cell Signaling Technology, 8690), p-JNK (Cell Signaling Technology, 9251), JNK (Cell Signaling Technology, 9252), β-actin (Santa Cruz, sc-47778), and GAPDH (Santa Cruz, sc-137179). The blots were visualized with chemiluminescence, and densitometric analysis of the blots was performed using an Amersham^™^ Imager 600 (E Healthcare, Chicago, IL, USA). Bands were semiquantitatively analyzed with ImageJ software, and the results are expressed as the ratio of the target proteins to the internal control gray value.

### Real-time PCR analysis

TRIzol reagent (AG, 21101) was used to extract total RNA from aortas and BMDMs, and the concentration and purity of the RNA were measured by a Thermo Scientific NanoDrop One. The reverse transcription reaction to synthesize cDNA was performed using a reverse transcription kit (AG, 11706), and the mRNA levels of inflammatory mediators were measured by a LightCycler^®^ 480 Instrument II using a SYBR Green real-time PCR premix kit (AG, 11701). The following primers were used: TNFα forward 5′-GTTCTATGGCCCAGACCCTCACA-3′, TNFα reverse 5′-TACCAGGGTTTGAGCTCAGC-3′; NLRP3 forward 5′-ATTACCCGCCCGAGAAAGG-3′, NLRP3 reverse 5′-TCGCAGCAAAGATCCACACAG-3′; IL-18 forward 5′-TCAAAGTGCCAGTGAACCCC-3′, IL-18 reverse 5′-GGTCACAGCCAGTCCTCTTAC-3′; IL-1β forward 5′-CTCGTGCTGTCGGACCCAT-3′, IL-1β reverse 5′-CAGGCTTGTGCTCTGCTTGTGA-3′; and β-actin forward 5′-ATCTGGCACCACACCTTC-3′, β-actin reverse 5′-AGCCAGGTCCAGACGCA-3′.

### Statistical analysis

Unpaired two-sided Student’s *t* tests or one-way ANOVA was used to calculate *P* values. A statistically significant difference was considered if *P* < 0.05 (NS, not significant). The data are expressed as the mean ± SEM. All experiments were repeated at least three times.

## Results

### DHC improves aortic compliance in ApoE^−/−^ mice fed a western diet

**Fig. 1:**
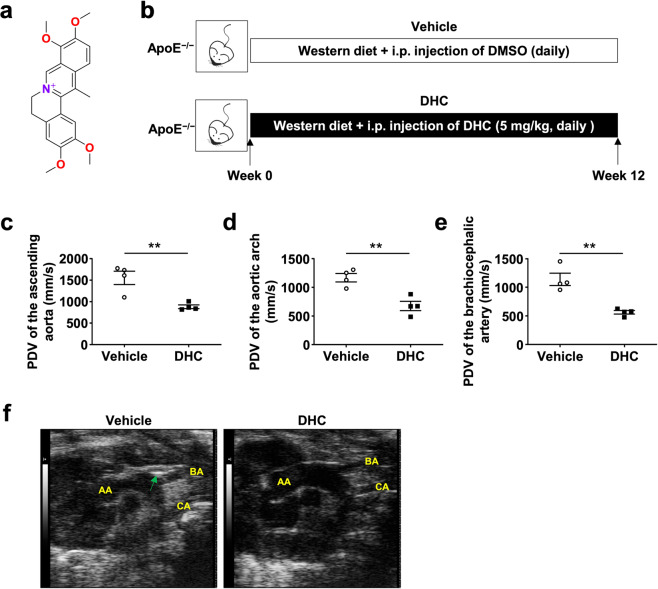
Dehydrocorydaline (DHC) improves aortic compliance in Western diet-fed ApoE^−/−^ mice. **a** Chemical structure of DHC (C_21_H_21_NO_4_). **b** Experimental protocol. Male 8-week-old ApoE^−/−^ mice were fed a western diet and intraperitoneally injected with vehicle or DHC (5 mg·kg^−1^·d^−1^) for 12 weeks. **c**–**e** Decreased peak flow velocity (PSV) of the ascending aorta (**c**), aortic arch (**d**), and brachiocephalic artery (**e**) in DHC-treated ApoE^−/−^ mice. *n* = 4 mice per group. **f** B-mode echocardiograms of the aorta, including the ascending aorta (AA), brachiocephalic artery (BA) branch, and carotid artery (CA), at 12 weeks after feeding. Green arrows indicate plaques. The data are shown as the mean ± SEM. **P < 0.01 vs. vehicle.

DHC (2,3,9,10-tetramethoxy-13-methyl-5,6-dihydroisoquinolino [2,1-] isoquinolin-7-ium) is a quaternary ammonium alkaloid, and the chemical structure of DHC is shown in Fig. 1a. To explore the effect of DHC on vascular function in vivo, Western diet-fed ApoE^−/−^ mice were treated intraperitoneally with vehicle or DHC (5 mg·kg^−1^·d^−1^) for 12 weeks (Fig. 1b). Murine aortas were assessed by ultrasonography as described in the Methods. After 12 weeks of DHC treatment, we observed decreased PDVs in the ascending aorta (Fig. 1c), aortic arch (Fig. 1d), and brachiocephalic artery (Fig. 1e), indicating that DHC improved aortic stiffness and vascular function in ApoE^−/−^ mice. In the B-mode echocardiograms of the aorta, we found that DHC reduced atherosclerotic plaque formation in the aortas of ApoE^−/−^ mice (Fig. 1f). Taken together, these data suggest that DHC may have vasoprotective effects and serve as an antiatherosclerotic agent.

### DHC attenuates atherosclerosis in ApoE^−/−^ mice

**Fig. 2:**
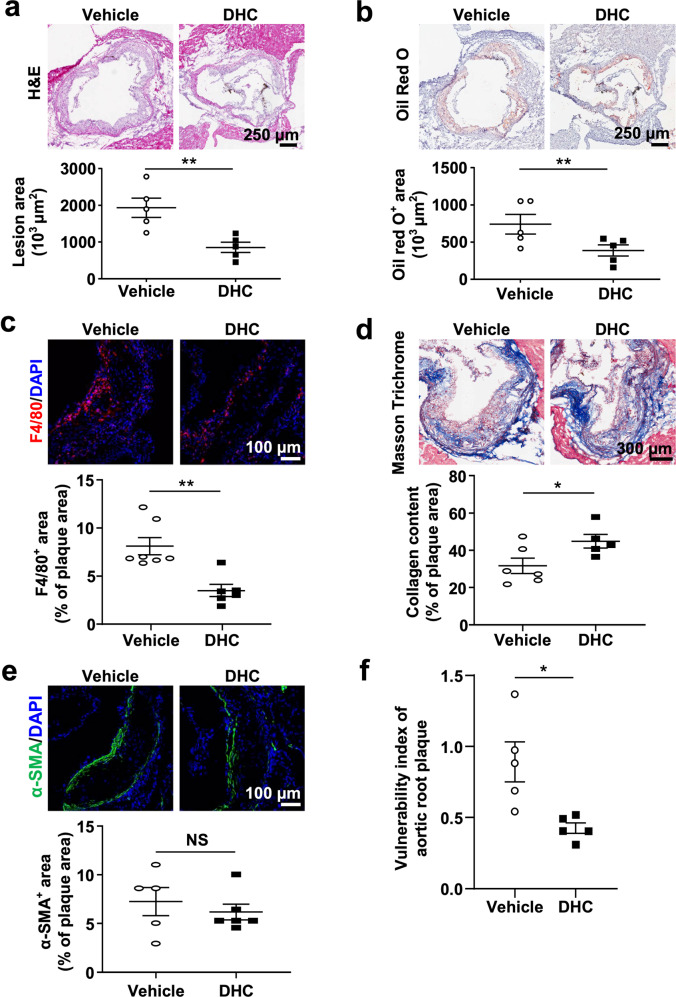
DHC attenuates atherosclerosis in ApoE^−/−^ mice fed a western diet for 12 weeks. **a** H&E-stained cross-sections of the aortic root. *n* = 5 mice per group. Scale bar, 250 μm. **b** Oil red O staining of aortas. *n* = 5 mice per group. Scale bar, 250 μm. **c** F4/80^+^ staining. *n* = 6–7 mice per group. Scale bar, 100 μm. **d** Masson’s trichrome staining. *n* = 5–6 mice per group. Scale bar, 300 μm. **e** α-SMA immunofluorescent staining of aortic root sections. *n* = 5–6 mice per group. Scale bar, 100 μm. **f** Quantitative analysis of plaque vulnerability indices in aortic plaques. *n* = 5 mice per group. The data are shown as the mean ± SEM. *P < 0.05, **P < 0.01 vs. vehicle. NS not significant.

To further validate the role of DHC in atherosclerosis, we directly visualized atherosclerotic lesions in the aortas of the abovementioned ApoE^−/−^ mice. Our imaging results showed that the atherosclerotic lesion area, as indicated by H&E staining, the oil red O^+^ area, and lesion macrophage levels (F4/80^+^), were significantly smaller in the aortic roots of DHC-treated ApoE^−/−^ mice than in control mice (Fig. 2a–c). In DHC-treated ApoE^−/−^ mice, collagen content was increased without significant alterations in smooth muscle cell numbers (Fig. 2d, e), and plaque vulnerability was markedly decreased (Fig. 2f). In addition, DHC treatment markedly decreased plasma TG levels but increased LDL-C levels (Table 1). However, no significant differences were observed in the plasma levels of HDL-C and TC or body weights between the vehicle- and DHC-treated groups (Table 1). Collectively, these results demonstrate that DHC exerts a protective effect against atherosclerosis.

**Table 1:** Effects of DHC on plasma lipids in ApoE^−/−^ mice fed with a western diet for 12 weeks.

	Vehicle	DHC (5 mg/kg)	*P*
TG (mmol/L)	1.8 ± 0.3	1.3 ± 0.2	0.0052**
TC (mmol/L)	23.1 ± 3.0	28.4 ± 5.1	0.0501
LDL-C (mmol/L)	8.3 ± 1.6	11.3 ± 1.9	0.0094**
HDL-C (mmol/L)	1.1 ± 0.3	1.5 ± 0.5	0.1596
Body weight (g)	32.1 ± 1.5	29.7 ± 2.7	0.0700

Data are presented as arithmetic means ± SEM. *n* = 7 mice per group.

*TC* total cholesterol, *LDL-C* low-density lipoprotein cholesterol, *HDL* high-density lipoprotein cholesterol, *TG* total triglycerides.

**P  <  0.01 vs. vehicle.

### DHC inhibits systemic and vascular inflammation in vivo

**Fig. 3:**
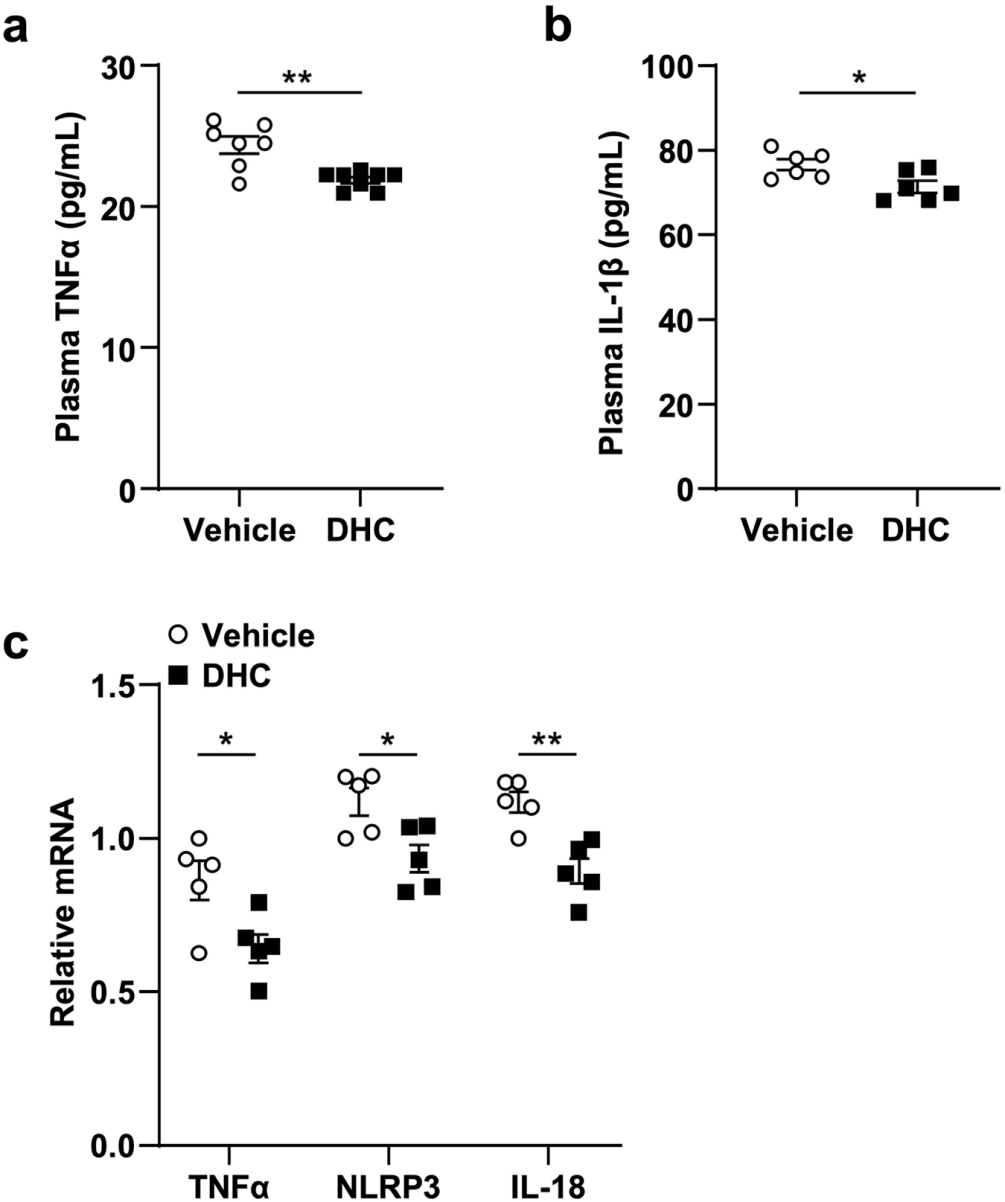
DHC reduces systemic and vascular inflammation in vivo. **a**, **b** Plasma levels of TNFα and IL-1β in Western diet-fed ApoE^−/−^ mice treated with vehicle or DHC for 12 weeks. *n* = 6–8 mice per group. **c** Relative mRNA levels of the proinflammatory genes TNFα, NLRP3, and IL-18 in aortas isolated from Western diet-fed ApoE^−/−^ mice treated with vehicle or DHC for 1 week. *n* = 5 mice per group. The data are shown as the mean ± SEM. *P < 0.05, **P < 0.01 vs. vehicle.

It is well accepted that inflammation plays a critical role in the development of atherosclerosis [20]. Since DHC plays a significant inhibitory role in atherosclerosis, we examined the impact of DHC on systemic and vascular inflammation in vivo. Compared to those of the vehicle, the ELISA results demonstrated that DHC treatment for 12 weeks markedly decreased plasma levels of TNFα and IL-1β (Fig. 3a, b). Furthermore, i.p. injection of DHC for 1 week significantly reduced TNFα, NLRP3, and IL-18 mRNA levels in the aortas of ApoE^−/−^ mice (Fig. 3c). Thus, these results suggest that DHC can inhibit aortic inflammation during atherosclerosis development.

### DHC inhibits inflammatory gene expression in BMDMs

**Fig. 4:**
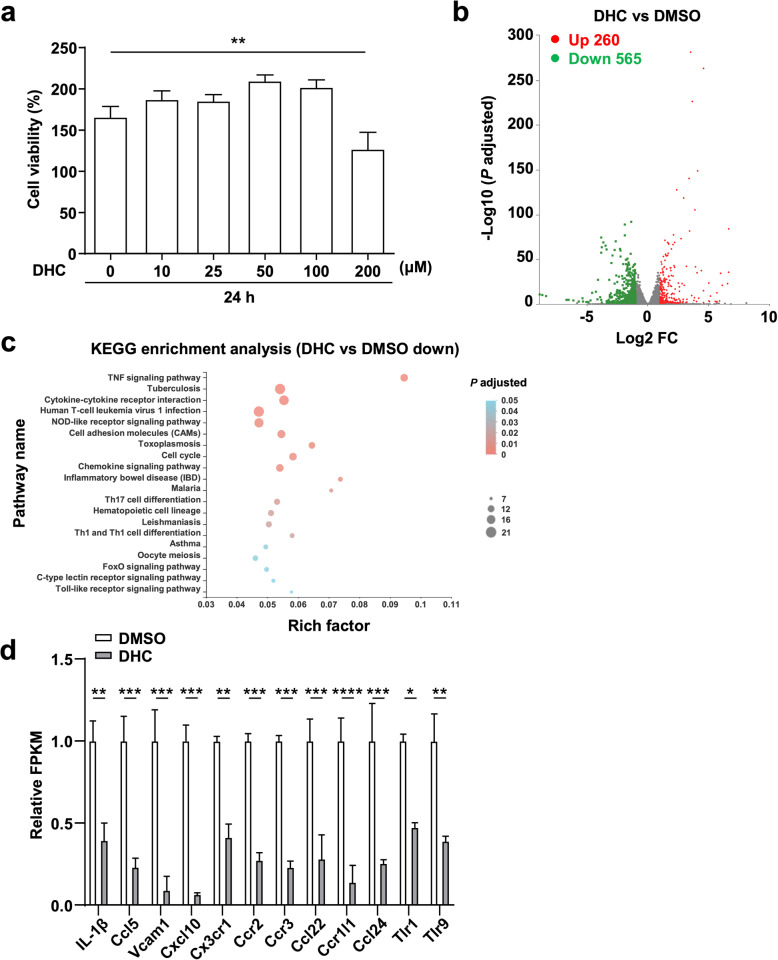
DHC downregulates inflammatory gene expression in BMDMs. **a** The toxicity of DHC on BMDMs was determined by CCK-8 assays. **b** A volcano plot of the RNA-seq data showing comparisons of BMDMs treated with DMSO or 100 μM DHC for 24 h. *n* = 3. Red and green dots indicate significantly up- and downregulated genes, respectively (adjusted *P* < 0.01). **c** Top 20 KEGG pathways for the downregulated differentially expressed genes (DEGs). The circle size represents the gene number. The color gradient shows the adjusted *P* value. **d** Bar graph showing the FPKM values of genes related to the inflammatory response. The data are shown as the mean ± SEM. *P < 0.05, **P < 0.01, ***P < 0.001, and ****P < 0.0001 vs. DMSO.

Since macrophages are the key players in atherosclerosis development, we then explored the effect of DHC on macrophages in vitro. First, we determined the toxicity of DHC in BMDMs. As shown in Fig. 4a, no cytotoxicity in BMDMs was observed for DHC at concentrations below 100 μM, while 200 μM DHC showed significant cytotoxicity, as indicated by the cell viability assay. Next, to gain a global transcriptional view of the effect of DHC treatment on macrophages, RNA-seq was conducted. The results showed that DHC (100 μM) differentially regulated 825 genes, including 260 upregulated genes and 565 downregulated genes (Fig. 4b). The 565 downregulated DEGs were mapped to KEGG pathways, and the top 20 enriched pathways are shown in Fig. 4c. These DEGs were highly clustered in several inflammation-associated signaling pathways, such as the TNF signaling pathway, cytokine-cytokine receptor interaction, tuberculosis, human T-cell leukemia virus 1 infection, the FoxO signaling pathway, malaria, and cell adhesion molecules. Importantly, inflammatory response genes, such as IL-1β, Ccl5, Vcam1, Cxcl10, Cx3cr1, Ccr2, Ccr3, Ccl22, Ccr1l1, Ccl24, Tlr1, and Tlr9, were significantly downregulated by DHC (Fig. 4d). Taken together, our data suggest that DHC may have a direct inhibitory effect on macrophage inflammation.

### DHC reduces inflammation in resting and activated BMDMs

**Fig. 5:**
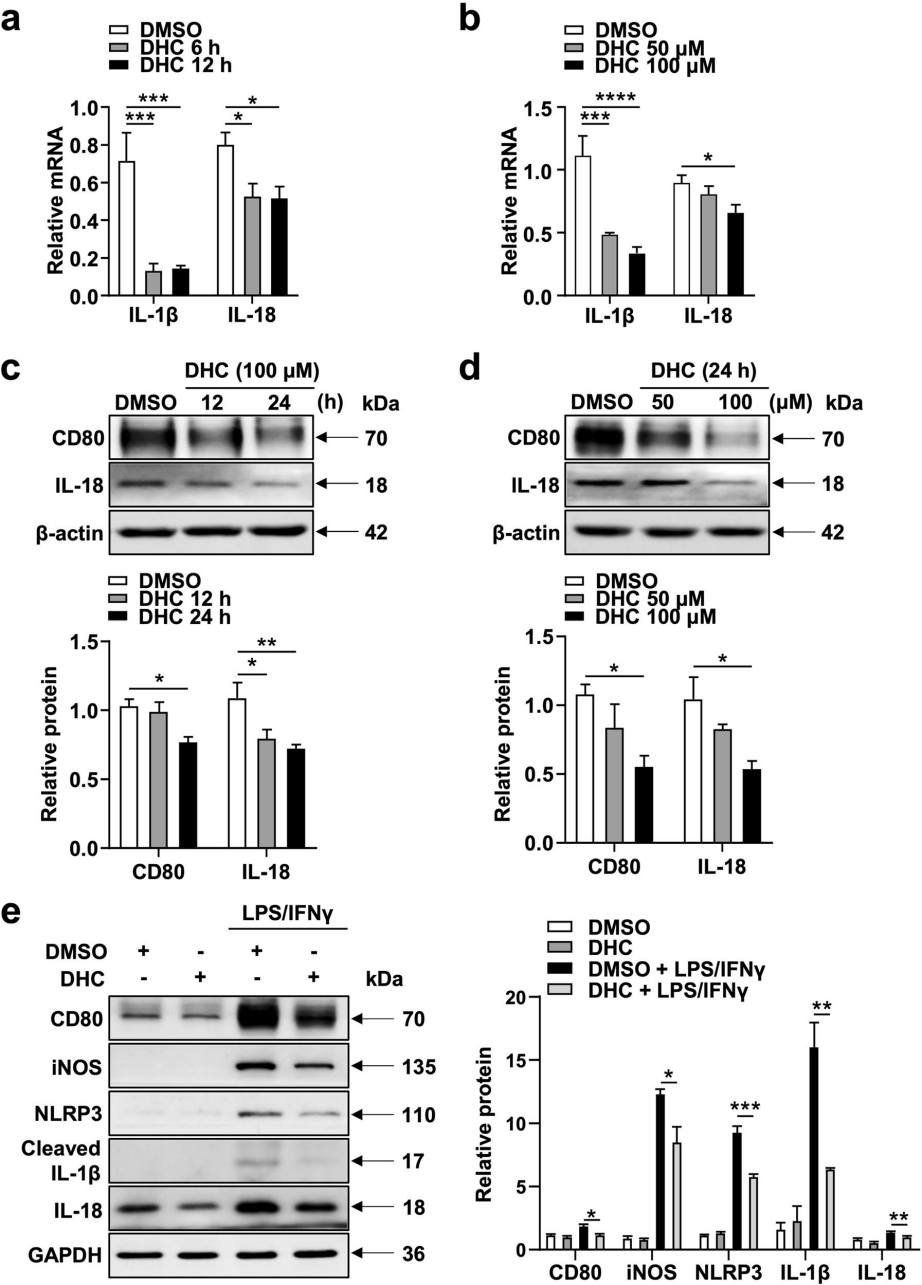
DHC alleviates inflammation in BMDMs. **a** Relative mRNA levels of IL-1β and IL-18 in BMDMs treated with 100 μM DHC for 0, 6, and 12 h. *n* = 3–5. **b** Relative mRNA levels of IL-1β and IL-18 in BMDMs treated with 0, 50, and 100 μM DHC for 12 h. *n* = 3–5. **c**–**d** Immunoblots of CD80 and IL-18 in BMDMs treated with DMSO or DHC as indicated. *n* = 3–4. **e** Immunoblots showing CD80, iNOS, NLRP3, IL-1β, and IL-18 in BMDMs induced with 100 ng/mL LPS- and 20 ng/mL IFNγ (LPS/IFNγ) and treated with DMSO or DHC for 24 h. *n* = 3–4. The data are shown as the mean ± SEM. *P < 0.05, **P < 0.01, ***P < 0.001, and ****P < 0.0001 vs. as indicated.

To further confirm the anti-inflammatory effect of DHC, we treated BMDMs with DMSO and DHC in the presence or absence of LPS and IFNγ (LPS/IFNγ). Consistent with our RNA-seq results, DHC dramatically decreased the mRNA levels of IL-1β and IL-18 in a time- and concentration-dependent manner in resting BMDMs (Fig. 5a, b). In addition, immunoblot analysis showed that DHC significantly downregulated the protein expression levels of CD80 and IL-18 in a time- and concentration-dependent manner in unstimulated BMDMs (Fig. 5c, d). Importantly, LPS and IFNγ dramatically upregulated proinflammatory protein levels (CD80, iNOS, NLRP3, IL-1β, and IL-18), which were significantly downregulated by DHC (Fig. 5e). Taken together, our data indicate that DHC exerts an anti-inflammatory effect on both resting and LPS/IFNγ-stimulated macrophages.

### DHC inhibits ERK1/2 and p65 activation in BMDMs

**Fig. 6:**
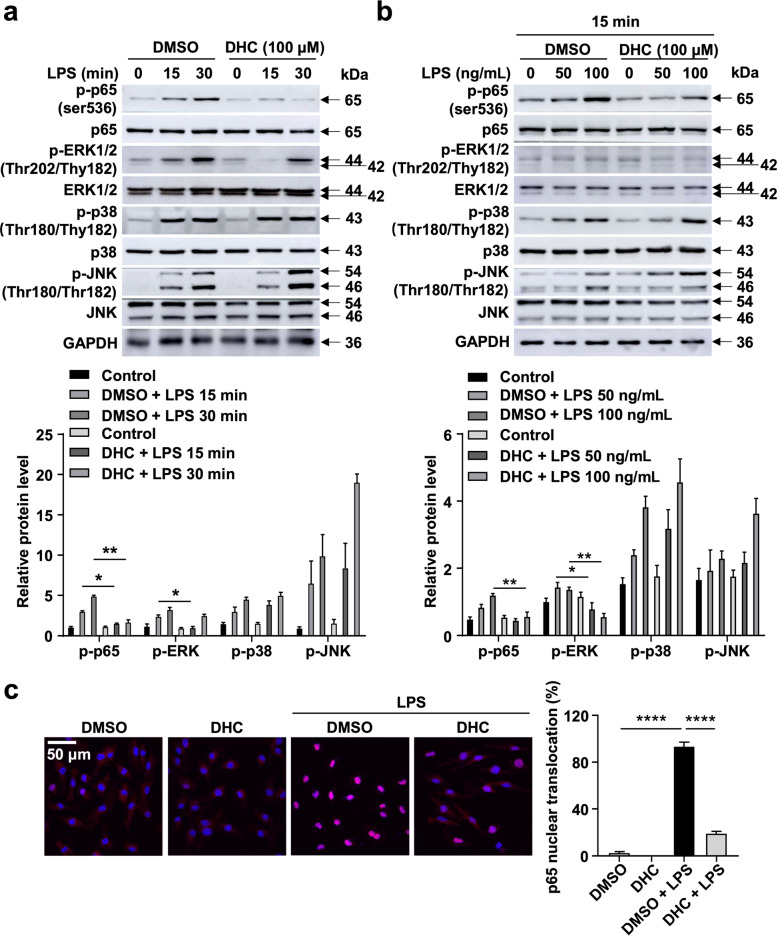
DHC limits p65 and ERK activation in BMDMs. **a** BMDMs were treated with 100 ng/mL LPS for 0, 15, or 30 min and incubated with DMSO or 100 μM DHC. *n* = 8. **b** BMDMs were treated with 0, 50, and 100 ng/mL LPS for 15 min and incubated with DMSO or 100 μM DHC. *n* = 5. **a**, **b** Immunoblots showing p-p65, p65, p-ERK1/2, ERK1/2, p-p38, p38, p-JNK, JNK, and GAPDH in the cells. **c** Immunofluorescent staining of p65 in BMDMs treated with DMSO or 100 μM DHC in the presence or absence of 100 ng/mL LPS for 15 min. *n* = 4. Scale bar, 50 μm. The data are shown as the mean ± SEM. *P < 0.05, **P < 0.01, ****P < 0.0001 vs. as indicated.

DHC significantly reduces the expression of proinflammatory mediators in macrophages, suggesting that DHC may attenuate inflammatory signaling pathways related to atherosclerosis. It has been well established that continuous activation of the nuclear factor-κB (NF-κB) [21] and mitogen-activated protein kinase (MAPK) [22, 23] signaling pathways in macrophages is involved in the development of atherosclerosis. We next explored whether DHC affected the NF-κB and MAPK inflammatory signaling pathways in macrophages. Thus, we stimulated BMDMs with LPS and then assessed the effects of DHC on LPS-induced activation of the NF-κB and MAPK signaling cascades. Our results showed that DHC treatment dramatically decreased p-p65 and p-ERK1/2 levels in a time- and concentration-dependent manner (Fig. 6a, b) but did not significantly affect p-p38 or p-JNK levels (Fig. 6a, b), suggesting that DHC specifically inhibits p65- and ERK1/2-associated pathways. Importantly, immunofluorescence analysis showed that LPS dramatically induced the nuclear translocation of p65, while DHC significantly inhibited LPS-induced p65 nuclear translocation in BMDMs (Fig. 6c). Therefore, these results indicate that DHC decreases macrophage inflammation by inhibiting p65- and ERK1/2-dependent signaling.

## Discussion

**Fig. 7:**
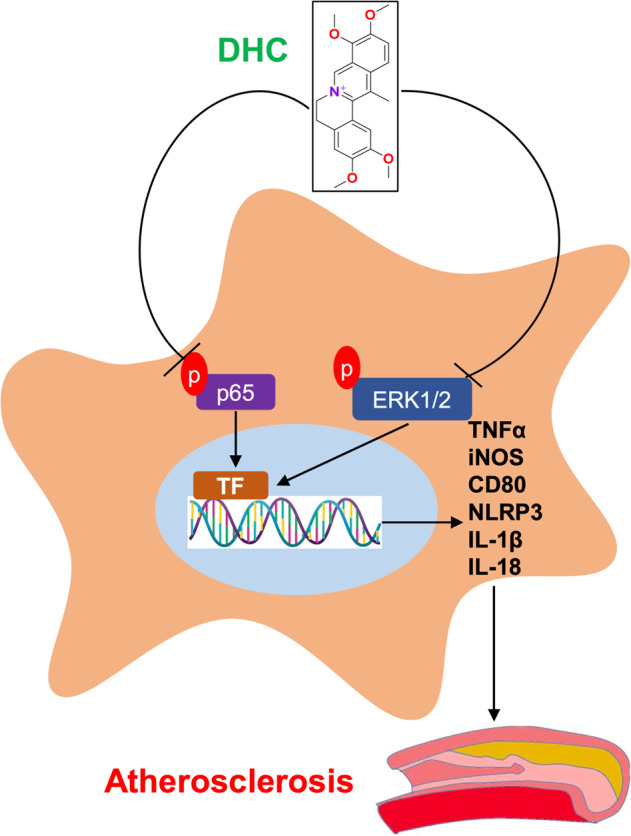
Model of the effects of DHC. By acting on macrophages, DHC inhibits the p65 and ERK1/2 pathways, leading to decreased expression of TNFα, iNOS, CD80, NLRP3, IL-1β, and IL-18. Consequently, DHC improves vascular function and attenuates atherosclerosis in western diet-fed ApoE^−/−^ mice.

Our current study has demonstrated for the first time that DHC ameliorates atherosclerosis in ApoE^−/−^ mice fed a Western diet, accompanied by improved aortic compliance and plaque stability. These beneficial effects of DHC on mice might be attributed to reductions in systemic and vascular inflammation. Using RNA-seq analysis, we demonstrated that DHC significantly downregulated multiple target genes involved in macrophage inflammatory activation. Then, we confirmed that DHC markedly reduced proinflammatory gene expression in BMDMs, likely by downregulating proinflammatory signals, such as p65 and ERK1/2 (Fig. 7).

The most important novel finding in this study was the antiatherosclerotic effect of DHC on atherosclerosis-prone ApoE^−/−^ mice. It has been reported that several alkaloids from plants protect against atherosclerosis and atherosclerotic CVDs. For example, berberine, an alkaloid extracted from the roots, rhizomes, and stems *of Coptis chinensis*, shows potent antiatherogenic effects on an atheroprone mouse model [8]. Another alkaloid, colchicine from *Colchicum autumnale*, decreases the risk of myocardial infarction, stroke, and the need for coronary revascularization in a broad spectrum of patients with coronary disease [24]. As an active alkaloid extracted from *Corydalis rhizoma*, DHC has been shown to reduce thrombin-induced platelet aggregation [25] and exerts protective effects against coronary heart diseases [15], including myocardial infarction [26]. For the first time, we reported that i.p. injection of DHC in cholesterol-enriched Western diet-fed ApoE^−/−^ mice dramatically ameliorated atherosclerotic lesion areas and promoted the stability of atherosclerotic plaques. Beneficial effects of DHC on vascular function were observed, as evidenced by improved aortic compliance. The newly found atheroprotective role of DHC in ApoE^−/−^ mice sheds light on the therapeutic application of this natural alkaloid in atherosclerosis treatment.

Given that inflammation plays a crucial role in the pathogenesis of atherosclerosis, blocking inflammation may prevent atherosclerosis. The preclinical CANTOS trial showed that IL-1β antibody treatment reduced recurrent cardiovascular events [9]. As our data demonstrated that DHC significantly reduced both systemic and vascular inflammation, it is likely that DHC inhibits atherosclerosis through its anti-inflammatory effects. This hypothesis has been strongly supported by our in vitro studies using DHC to treat BMDMs. First, our RNA-seq analysis showed that DHC significantly inhibited macrophage activation, as evidenced by the downregulation of proinflammatory gene clusters. Second, DHC not only reduced basal inflammation but also inhibited LPS/IFNγ-induced inflammation in BMDMs. Notably, DHC markedly reduced LPS/IFNγ-induced NLRP3, IL-1β, and IL-18 protein levels in BMDMs, indicating a specific inhibitory effect of DHC on the activation of NLRP3 inflammation. Consistent with our findings, inhibition of the NLRP3 inflammasome by the selective inhibitor MCC950 reduces atherosclerosis in ApoE^−/−^ mice [27]. Next, we showed that DHC alleviated LPS-induced p65 and ERK1/2 activation but not p38 or p-JNK activation. Consistent with our work, DHC downregulates MEK1/2-ERK1/2 cascades in melanoma cells and inhibits cell proliferation, migration, and invasion [28]. Recently, Kong et al. reported that DHC decreased inflammatory cytokine expression and release by promoting IκBα expression, suppressing activation of the NF-κB transcriptional element and reducing the nuclear translocation of NF-κB [29]. The effects of DHC on p38 MAPK seem to vary among cell types. In our system, DHC did not markedly affect p38 phosphorylation. However, DHC stimulates p38 MAPK activation and promotes cell differentiation in myoblasts [30]. The fine regulation of inflammation activation signals in different cell types by DHC requires further investigation.

Although we observed potent anti-inflammatory effects of DHC, how DHC exerts its inhibitory effect on macrophage inflammation or inhibits the expression of proinflammatory genes remains unclear. Different alkaloids may exert their anti-inflammatory effects via distinct mechanisms. For example, colchicine exerts its anti-inflammatory effects by inhibiting microtubule polymerization and repressing the Ras homolog gene family member A (RhoA)/Rho-associated coiled-coil containing protein kinase (ROCK) pathway [31]. In addition, berberine exerts anti-inflammatory effects through inhibition of the MAPKs, AMP-activated protein kinase, and NF-κB pathways [32]. Previously, two independent studies demonstrated that DHC has potent acetylcholinesterase (AChE) inhibitory activities [33, 34]. Evidence has shown that AChE promotes inflammation by both directly hydrolyzing acetylcholine (ACh) and interacting with the α7 nicotinic ACh receptor (α7 nAChR) [35]. Therefore, the potential mechanism of the anti-inflammatory effect of DHC may be related to AChE. However, whether DHC exerts an anti-inflammatory effect by inactivating AChE warrants further exploration.

It is possible that DHC may exert antiatherosclerotic effects via multiple mechanisms. As indicated by our results, DHC decreases blood levels of “bad” TG. Accumulating evidence suggests that TG-rich lipoproteins are causal factors in atherogenesis [7], and lowering serum TG-rich lipoprotein levels may be an effective approach for treating atherosclerosis. However, it should also be noted that DHC increases blood levels of “bad” LDL-C. Increased LDL-C is detrimental to atherosclerosis. There are two possibilities for the antiatherosclerotic effects of DHC in vivo. First, the beneficial effects of DHC on blood lipids exceed its detrimental effects. Second, the anti-inflammatory role of DHC is dominant during atherosclerosis development.

In conclusion, the results of this study on BMDMs and ApoE^−/−^ mice suggest that DHC reduces atherosclerosis and inflammation by inhibiting the activation of p65 and ERK1/2, and the natural product DHC may be a potential drug to treat atherosclerosis and its associated sterile inflammation.
